# Recurrent CIC-rearranged sarcoma of central nervous system: a clinicopathological case report

**DOI:** 10.3389/fonc.2025.1545700

**Published:** 2026-01-26

**Authors:** Shan Jiang, Kay Ka-Wai Li, Jingqi Hu, Yun Guan, Rui-Ze Zhu, Hou-Shi Xu, Mao-Yuan Sun, Bei-Ning Liu, Hui-Hui Chai, Yue Wang, Qi-Sheng Tang, Ho-Keung Ng, Zhi-Feng Shi

**Affiliations:** 1 Department of Neurosurgery, Huashan Hospital, Fudan University, Shanghai, China; 2 National Center for Neurological Disorders, Shanghai, China; 3 Neurosurgical Institute, Fudan University, Shanghai, China; 4 Shanghai Clinical Medical Center of Neurosurgery, Shanghai, China; 5 Department of Anatomical and Cellular Pathology, The Chinese University of Hong Kong, Hong Kong, Hong Kong SAR, China

**Keywords:** case report, CIC rearranged sarcoma, CyberKnife, methylation classifiers, MTB

## Abstract

CIC-rearranged sarcoma (CRS) is a subgroup of small round blue cell tumors similar to Ewing’s sarcoma but lacking the EWSR1 gene translocation. It is characterized by a CIC gene rearrangement on chromosome 19q13, with the most common partner gene being DUX4. In 2021, the WHO classification of central nervous system tumors (5th edition) introduced a new pathological classification for CRS based on bone and soft tissue tumor classifications, categorizing it as mesenchymal non-meningeal epithelial tumors. Primary CIC::DUX4 rearrangement sarcoma in the central nervous system is extremely rare, with no established standard treatment and a very poor prognosis. Herein, we present a case of intracranial CIC::DUX4 rearrangement diagnosed using DNA methylation classifiers and multiple omics diagnosis techniques. We also document the disease progression after chemoradiation therapy and subsequent *in situ* recurrence.

## Case report

**Figure 1:**
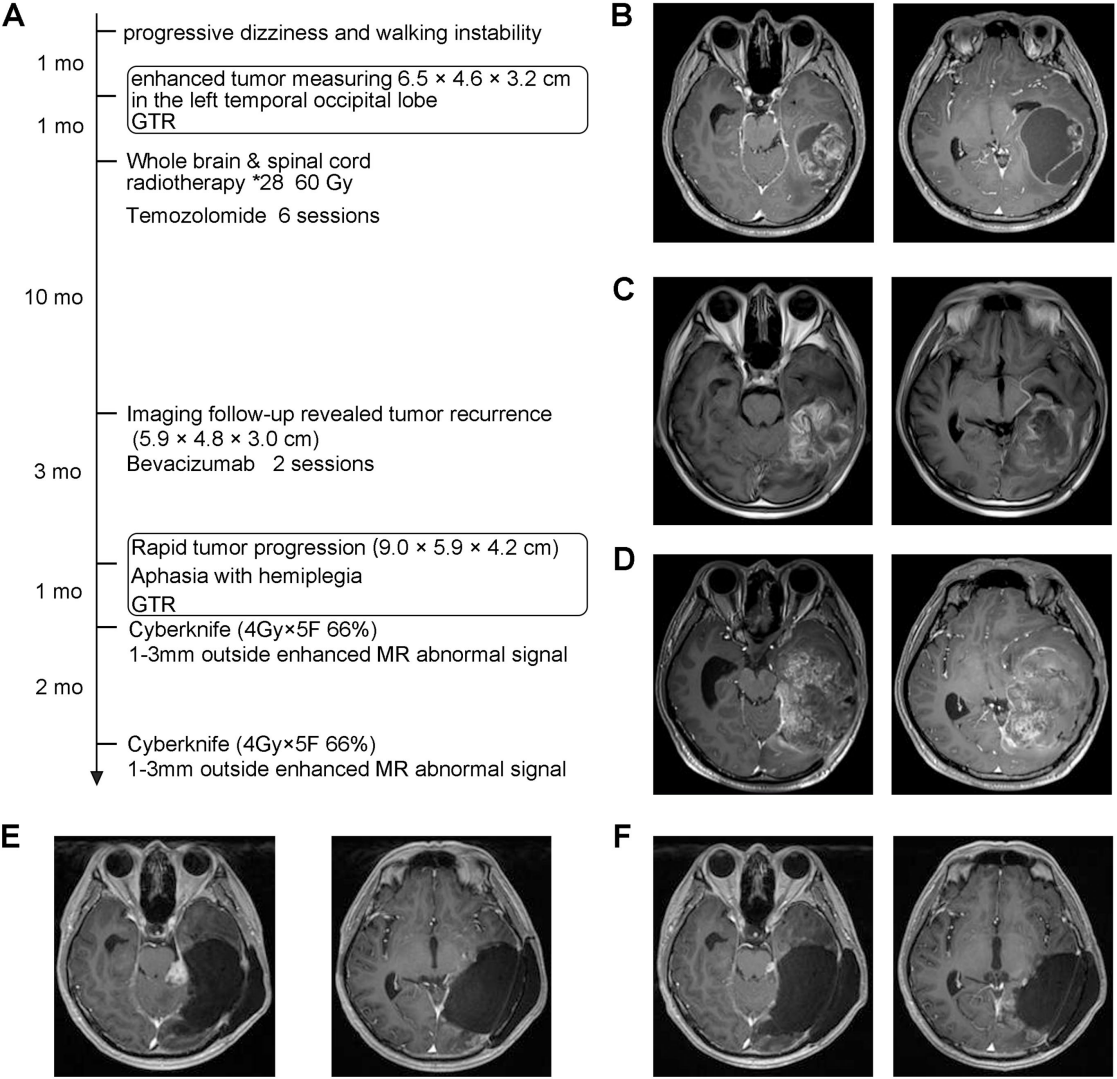
Diagnosis and treatment process of the case. **(A)** Patient treatment timeline. **(B)** Representative T1-weighted contrast enhanced MR images before the first operation. **(C)** T1 enhanced MRI representative images at the first postoperative imaging follow-up at 11 months. **(D)** Representative T1 enhanced MR images before the second operation. **(E)** T1 enhanced MRI representative image before the first CyberKnife treatment. **(F)** T1 enhanced MRI representative image before the second CyberKnife treatment.

A 41-year-old man presented with progressive exacerbation of dizziness and walking instability persisting for over 1 month (**Figure 1A**). There was no family history of tumor disease or known hereditary genetic disorders. Enhanced MRI revealed the presence of an enhanced tumor measuring 6.5 × 4.6 × 3.2 cm in the left temporal occipital lobe, which was subsequently surgically resected (**Figure 1B**). Postoperative pathology confirmed the diagnosis as “round cell malignant tumor, initially considering embryonic tumor of the central nervous system, NOS, WHO Grade 4”. One month after discharge, the patient underwent whole-brain and spinal irradiation (60 Gy) along with seven cycles of temozolomide adjuvant chemotherapy (5/28 regimen), and then, he received regular clinical follow-up.

At 11 months after the initial surgery, MRI indicated recurrence of the tumor without any accompanying symptoms. PET–CT examination showed no tumor lesions in other parts of the body except the central nervous system (**Figure 1C**). Two cycles of bevacizumab combined with temozolomide (5/28 regimen) were administered as treatment for this recurrence. Three months later, enhanced MR showed progressive disease, and the patient suffered from a new onset of right-sided hemiplegia accompanied by aphasia. A second tumor resection surgery was performed (**Figure 1D**).

## Neuropathological findings

**Figure 2:**
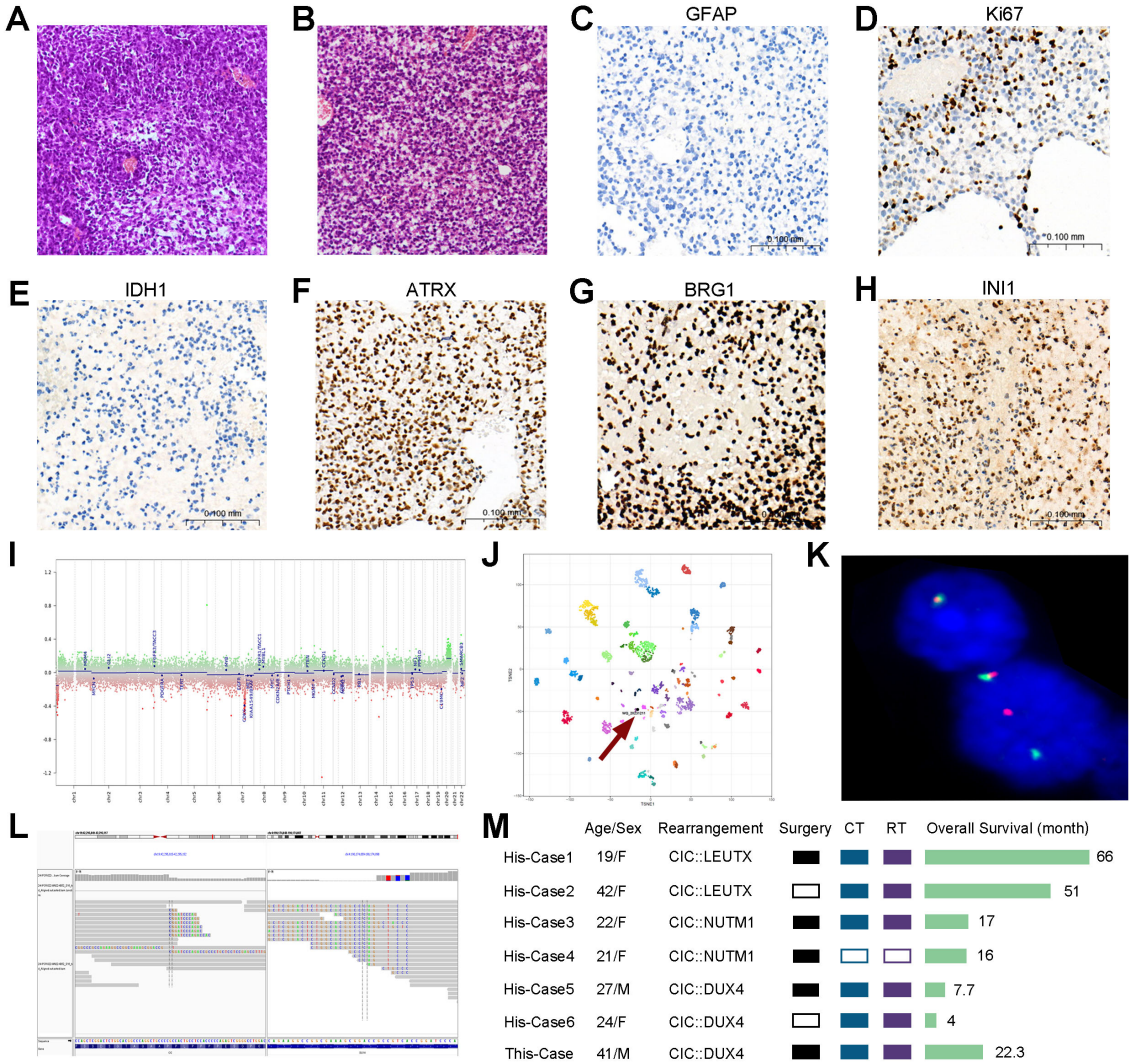
Diagnosis and treatment process of the case. **(A)** H&E section of the primary tumor (×200). **(B)** H&E section of the recurrent tumor (×200). **(C–H)** Representative IHCs of the recurrent tumor. Scar bar, 100 μm. **(I)** Copy number variations of relapsed tumor according to DNA methylation profiling. **(J)** Unsupervised clustering by t-SNE of DNA methylation profiling showed a designation of sarcoma. **(K)** Break Apart FISH: tumor cells were detected to have a translocation of the CIC gene (Zytolight, ZyGreen labeled with chr19:42,835,047–43,374,575 distal to the CIC breakpoint region, ZyOrange labeled with chr19:41,980,301–42,751,339 proximal to the CIC breakpoint region). **(L)** RNA sequencing showed that exon 20 of the CIC gene was fused with exon 1 of DUX4 gene. **(M)** Previously reported adult cases of supratentorial CIC-rearranged sarcoma (His-case 1–6 (2–7) in order) compared with this reported case. Ct, chemotherapy; RT, radiotherapy. Only cases where the diagnosis identified specific rearrangement of partner genes were included.

The morphological and immunohistochemical staining characteristics of the primary and recurrent tumors were similar. Histology exhibited medium-sized to large round cells with hyperchromatic and pleomorphic nuclei. Mucoid interstitium was often seen, along with extensive regional pattern necrosis (**Figures 2A, B**). Immunohistochemical staining revealed high expression of Ki67 (more than 50%), while it was negative for IDH1 and GFAP, but ATRX retained nuclear expression (**Figures 2C–F**). Targeted-panel next-generation sequencing (NGS) was performed on the primary tumor sample. The detection range covered all coding regions of 528 genes, partial regions of 154 genes, and three virus sequences. The types of mutations detected included base replacement (SNV), insertion–deletion of small fragments (Indel), copy number variation (CNV), and gene fusion. NGS was based on Illumina and achieved 20,000× ctDNA deep sequencing. Based on NGS detection results combined with algorithm analysis, the tumor sample had no detected deletions of 1p19q or amplification/undeletion events involving chromosome 7/10, EGFR amplification, or BRAF fusion mutation. Moreover, biallelic mutation of SMARCB1/SMARCA4 was detected, which was consistent with IHCs of INI1 and BRG1 (**Figures 2G, H**). No mutations were found in the DICER1 and BCOR genes. It is worth mentioning that EWSR or CIC variants were not detected in the targeted-panel NGS. The CNV in the panel region was not detected.

Despite combining NGS with targeted tumor gene panels, a definitive pathological diagnosis could not be obtained, leading to the exclusionary diagnosis known as central nervous system (CNS) embryonal tumor NOS. To address this issue, we employed 850K methylation chips to analyze fresh frozen samples of the recurrent tumor and uploaded the IDAT file to the DKFZ classifier v12.8 (Molecular Neuropathology, Heidelberg, Germany) (1), and a diagnosis of CIC-rearranged sarcoma was established. The chromosomal CNV profile generated from raw methylation data did not demonstrate any large-scale alterations (**Figures 2I, J**). We validated the classifier’s findings by RNA sequencing (RNA-seq) and FISH test with SPEC ClC Dual Color Break Apart Probe (**Figure 2K**). We performed RNA-seq on paraffin tissue from both primary and recurrent tumors by STAR-Fusion-v1.9.1 analysis, and it confirmed the presence of CIC::DUX4 fusion in both samples, indicating that exon 20 of the CIC gene fused with exon 1 of the DUX4 gene (**Figure 2L**).

## Treatment and clinical outcome

CIC-rearranged sarcoma is an extremely rare adult CNS neoplasm, with only a few cases reported in the literature (**Figure 2M**). Currently, there is no standardized treatment for CIC-rearranged sarcoma of the CNS. One month after the second surgical resection, imaging follow-up revealed a high signal in the enhanced T1 image near the hippocampus, and the area with abnormal signal on the enhanced MR was treated with 1–3-mm CyberKnife (4 Gy × 5 F 66%). After 2 months, a significant reduction of abnormal signal was observed, leading to a second CyberKnife treatment (4 Gy × 5 F 70%). No notable impairment of cognitive, motor abilities, and linguistic function was observed following CyberKnife treatment, and there was a substantial decrease in the abnormal signal area on imaging. At present, no tumor recurrence has been detected during imaging follow-up, and self-care capabilities have been maintained. The patient’s survival time since the first surgery was recorded as 22.0 months so far, and no tumor recurrence was found at the time of submission.

## Discussion

In 2016, Sturm and colleagues conducted a comprehensive genomic and methylation identification of 323 tumors diagnosed with primary CNS-PNET at multiple collaborating institutions, demonstrating that “PNET” was biologically heterogeneous rather than a single tumor type (8). CIC-rearranged sarcoma has therefore been classified as a separate subtype and incorporated into the 5th edition of the WHO classification of CNS tumors. CIC-rearranged sarcoma represents a subgroup of small round blue cell tumors resembling Ewing’s sarcoma, but lacking EWSR1 gene translocation. It is characterized by a CIC gene rearrangement located on chromosome 19q13. In this case report, we employed various molecular techniques, including methylation profiling, RNA-seq, and FISH staining, to confirm the rare diagnosis of supratentorial CIC::DUX4 rearrangement sarcoma.

Although the histopathological features of CIC-rearranged sarcoma closely resemble those of Ewing’s sarcoma, its molecular characteristics and clinical manifestations differ significantly. CIC-rearranged sarcoma of the soft tissues exhibits high invasiveness and frequently presents with metastasis, most commonly to the lungs (9). Notably, the overall survival rate is lower compared to that of Ewing’s sarcoma and other undifferentiated small round cell sarcomas (6). A review encompassing 88 cases of systemic CIC-rearranged sarcomas demonstrated a significantly lower 3-year survival rate (39.5%) in patients with CIC-rearranged sarcomas compared to other undifferentiated small round cell sarcomas (78.7%) and BCOR::CCNB3-associated tumors (78.7%) (10). In six reported cases, the CIC::DUX4 rearrangement subtype had a worse prognosis than other types, while two cases with the CIC::LEUTX subtype combined with temozolomide (TMZ) chemotherapy and radiotherapy showed significantly longer survival. In light of the lack of standard therapeutic approaches for this condition, our case report provides a comprehensive diagnosis and treatment for primary CNS CIC-rearranged sarcoma from initial presentation through recurrence, highlighting a favorable response following postoperative CyberKnife treatment after tumor recurrence. In this case, the methylation classifier diagnosis aided in resolving challenging diagnoses for rare CNS tumor subtypes. This approach overall enhances diagnostic accuracy for rare CNS tumors.

## Data Availability

The raw data supporting the conclusions of this article will be made available by the authors, without undue reservation.
