# Supplementary material for: Recurrent CIC-rearranged sarcoma of central nervous system: a clinicopathological case report
Source: Front Oncol. 2026 Jan 26;15:1545700. doi: 10.3389/fonc.2025.1545700 (PMC12883352; doi:10.3389/fonc.2025.1545700)
Supplement: Supplementary file 1 [file DataSheet1.pdf]

A

ABCB1(NM_000927)	*ACTL6A*(NM_004301)	*ALDH2*(NM_000690)	*AREG*(NM_001657)	*ATR*(NM_001184)
ABCC1(NM_004996)	*ACVR1*(NM_001105)	*ALK*(NM_004304)	*ATRIP*(NM_130384)	*ARID1A*(NM_006015)
ABCC11(NM_032583)	*ADCY2*(NM_020546)	*ALOX12*(NM_000697)	*ARID1B*(NM_020732)	*ATRX*(NM_000489)
ABCC2(NM_000392)	*ADH1B*(NM_000668)	*AMER1*(NM_152424)	*ARID2*(NM_152641)	*AURKA*(NM_003600)
ABCC4(NM_005845)	*ADH1C*(NM_000669)	*ANXA5*(NM_001154)	*AURKB*(NM_004217)	*ARMS2*(NM_001099667)
ABCC5(NM_005688)	*AIP*(NM_003977)	*APC*(NM_000038)	*ASNS*(NM_001673)	*AXIN1*(NM_003502)
ABCG1(NM_016818)	*AXIN2*(NM_004655)	*APLF*(NM_173545)	*ASPH*(NM_004318)	*AKR1C3*(NM_001253908)
ABCG2(NM_004827)	*AKT1*(NM_005163)	*APOB*(NM_000384)	*ASXL1*(NM_015338)	*AXL*(NM_001699)
ABL1(NM_005157)	*AKT2*(NM_001626)	*AR*(NM_000044)	*ATM*(NM_000051)	
ACSS2(NM_018677)	*AKT3*(NM_181690)	*ARAF*(NM_001654)	*ATP7B*(NM_000053)	

B

B2M(NM_004048)	*BCL2L1*(NM_138578)	*BCORL1*(NM_021946)	*BRCA1*(NM_007294)	*BRE*(NM_004899)
BAP1(NM_004656)	*BCL2L11*(NM_138625)	*BLM*(NM_000057)	*BRCA2*(NM_000059)	*BRIP1*(NM_032043)
BARD1(NM_000465)	*BCL6*(NM_001706)	*BMPR1A*(NM_004329)	*BRD2*(NM_001113182)	*BTK*(NM_000061)
BCL2(NM_000633)	*BCOR*(NM_001123383)	*BRAF*(NM_004333)	*BRD4*(NM_058243)	*BUB1*(NM_004336)

C

C8orf34(NM_052958)	*CCL18*(NM_002988)	*CDK8*(NM_001260)	*CLK2*(NM_003993)	*CXXC4*(NM_025212)
CACNA1C(NM_000719)	*CCND1*(NM_053056)	*CDKN1A*(NM_078467)	*CMPK1*(NM_016308)	*CYLD*(NM_015247)
CADM2(NM_153184)	*CCND2*(NM_001759)	*CDKN1B*(NM_004064)	*CYP19A1*(NM_000103)	*CNTNAP5*(NM_130773)
CALR(NM_004343)	*CCND3*(NM_001760)	*CDKN1C*(NM_000076)	*CREBBP*(NM_004380)	*CYP1A1*(NM_000499)
CAMTA1(NM_015215)	*CCNE1*(NM_001238)	*CDKN2A*(NM_000077)	*CRKL*(NM_005207)	*CYP1A2*(NM_000761)
CAPN2(NM_001748)	*CD274*(NM_014143)	*CDKN2B*(NM_004936)	*CRLF2*(NM_022148)	*CYP1B1*(NM_000104)
CARD11(NM_032415)	*CD74*(NM_001025159)	*CDKN2C*(NM_001262)	*CSDE1*(NM_001007553)	*CYP2B6*(NM_000767)
CASC8(NR_024393)	*CD79A*(NM_001783)	*CEBPA*(NM_004364)	*CSF1R*(NM_005211)	*CYP2C19*(NM_000769)
CASP7(NM_001227)	*CD79B*(NM_001039933)	*CES1*(NM_001025195)	*CSF3R*(NM_156039)	*CYP2C8*(NM_000770)
CASP8(NM_001228)	*CDA*(NM_001785)	*CFD*(NM_001928)	*CSMD3*(NM_052900)	*CYP2C9*(NM_000771)
CBFB(NM_001755)	*CDC25C*(NM_001790)	*CFH*(NM_000186)	*CSNK1A1*(NM_001892)	*CYP2D6*(NM_000106)
CBL(NM_005188)	*CDC73*(NM_024529)	*CFTR*(NM_000492)	*CSNK2A1*(NM_001895)	*CYP2E1*(NM_000773)
CBLB(NM_170662)	*CDH1*(NM_004360)	*CHD4*(NM_001273)	*CTCF*(NM_006565)	*CYP3A4*(NM_017460)
CBR1(NM_001757)	*CDK12*(NM_016507)	*CHEK1*(NM_001274)	*CTNNA1*(NM_001903)	*CYP3A5*(NM_000777)
CBR3(NM_001236)	*CDK2*(NM_001798)	*CHEK2*(NM_007194)	*CTNNB1*(NM_001904)	
CCAT2(NR_109834)	*CDK4*(NM_000075)	*FNTB*(NM_002028.3)	*CUL3*(NM_003590)	
CCDC6(NM_005436)	*CDK6*(NM_001259)	*CIC*(NM_015125)	*CXCR4*(NM_003467)	

D

DAXX(NM_001141969)	*DDR2*(NM_006182)	*DHFR*(NM_000791)	*DNMT3A*(NM_022552)	*DSCAM*(NM_001389)
DDB2(NM_000107)	*DDX3X*(NM_001356)	*DICER1*(NM_177438)	*DOT1L*(NM_032482)	*DYNC2H1*(NM_001080463)
DDIT3(NM_004083)	*DDX51*(NM_175066)	*DNMT1*(NM_001130823)	*DPYD*(NM_000110)	

E

E2F7(NM_203394)	*EML4*(NM_019063)	*EPHB1*(NM_004441)	*ERCC5*(NM_000123)	*ETV6*(NM_001987)

ECT2L(NM_001077706)	ENOSF1(NM_017512)	EPHX1(NM_001136018)	ERCC6(NM_000124)	EWSR1(NM_005243)
EDN1(NM_001955)	EP300(NM_001429)	ERBB2(NM_004448)	EREG(NM_001432)	EXT1(NM_000127)
EED(NM_003797)	EPAS1(NM_001430)	ERBB3(NM_001982)	ERG(NM_182918)	EXT2(NM_000401)
EEF1A1(NM_001402)	EPCAM(NM_002354)	ERBB4(NM_001042599)	ERRFI1(NM_018948)	EZH2(NM_004456)
EGF(NM_001963)	EPHA2(NM_004431)	ERCC1(NM_001983)	ESR1(NM_000125)	
EGFR(NM_005228)	EPHA3(NM_005233)	ERCC2(NM_000400)	ESR2(NM_001040275)	
EIF3A(NM_003750)	EPHA5(NM_004439)	ERCC3(NM_000122)	ETV1(NM_004956)	
ELAC2(NM_001165962)	EPHA7(NM_004440)	ERCC4(NM_005236)	ETV4(NM_001986)	

F

FAM175A(NM_139076)	FAT1(NM_005245)	FGF23(NM_020638)	FGFR2(NM_000141)	FOXA1(NM_004496)
FAM46C(NM_017709)	FBN3(NM_032447)	FGF3(NM_005247)	FGFR3(NM_000142)	FOXK2(NM_004514)
FANCA(NM_000135)	FBXW7(NM_018315)	FGF4(NM_002007)	FGFR4(NM_022963)	FOXL2(NM_023067)
FANCB(NM_001018113)	FCGR2A(NM_021642)	FGF5(NM_004464)	FH(NM_000143)	FOXM1(NM_021953)
FANCC(NM_000136)	FCGR3A(NM_001127593)	FGF6(NM_020996)	FLCN(NM_144997)	FOXP1(NM_032682)
FANCG(NM_004629)	FGF1(NM_000800)	FGF7(NM_002009)	FLT1(NM_002019)	FOXP2(NM_014491)
FANCI(NM_001113378)	FGF10(NM_004465)	FGF8(NM_006119)	FLT3(NM_004119)	FRS2(NM_006654)
FANCL(NM_018062)	FGF19(NM_005117)	FGF9(NM_002010)	FLT4(NM_182925)	FUBP1(NM_003902)
FANCM(NM_020937)	FGF2(NM_002006)	FGFR1(NM_023110)	FOLR3(NM_000804)	FUS(NM_004960)

G

GAB2(NM_080491)	GATA6(NM_005257)	GLIPR1(NM_006851)	GPER1(NM_001039966)	GSK3B(NM_001146156)
GALNT12(NM_024642)	GEMIN6(NM_024775)	GLRX(NM_002064)	GPRIN2(NM_014696)	GSR(NM_000637)
GALNT14(NM_001253826)	GEN1(NM_182625)	GMEB1(NM_006582)	GPX5(NM_001509)	GSTA1(NM_145740)
GATA1(NM_002049)	GGH(NM_003878)	GNA11(NM_002067)	GREM1(NM_013372)	GSTM3(NM_000849)
GATA2(NM_032638)	GK5(NM_001039547)	GNAQ(NM_002072)	GRIN2A(NM_000833)	GSTP1(NM_000852)
GATA3(NM_001002295)	GLI1(NM_005269)	GNAS(NM_000516)	GRM1(NM_001278064)	

H

H19(NR_002196)	HGF(NM_000601)	HLA-DPB1(NM_002121)	HNF1A(NM_000545)	HSD17B3(NM_000197)
H3F3A(NM_002107)	HIF1A(NM_001530)	HLA-DQB1(NM_002123)	HNF1B(NM_000458)	HSD3B2(NM_000198)
HDAC2(NM_001527)	HKDC1(NM_025130)	HLA-DRB1(NM_002124)	HOTAIR(NR_047517)	HSP90AA1(NM_005348)
HERC2(NM_004667)	HLA-A(NM_002116)	HLA-G(NM_002127)	HOXB13(NM_006361)	HSPA5(NM_005347)
HFE2(NM_145277)	HLA-B(NM_005514)	HMGA2(NM_003483)	HPSE2(NM_021828)	HTRA1(NM_002775)
HFM1(NM_001017975)	HLA-C(NM_002117)	HMGCR(NM_000859)	HRAS(NM_005343)	

I-J

IDH1(NM_005896)	IFNLR1(NM_170743)	IKZF1(NM_006060)	IL7R(NM_002185)	IRS2(NM_003749)
IDH2(NM_002168)	IGF1R(NM_000875)	IL13(NM_002188)	INHBA(NM_002192)	JAK1(NM_002227)
IFNGR1(NM_000416)	IGF2(NM_000612)	IL16(NM_001172128)	INPP4B(NM_003866)	JAK2(NM_004972)
IFNGR2(NM_005534)	IGFBP3(NM_001013398)	IL1B(NM_000576)	IRF1(NM_002198)	JAK3(NM_000215)
IFNL2(NM_172138)	IKBKE(NM_001193321)	IL23R(NM_144701)	IRF4(NM_001195286)	JUN(NM_002228)

K

KCNJ5(NM_000890)	KDR(NM_002253)	KIT(NM_000222)	KMT2A(NM_001197104)	KRAS(NM_004985)
KDM5A(NM_001042603)	KEAP1(NM_012289)	KLC1(NM_001130107)	KMT2B(NM_014727)	KRT14(NM_000526)
KDM5C(NM_004187)	KIF1B(NM_015074)	KLF4(NM_004235)	KMT2C(NM_170606)	KRT15(NM_002275)
KDM6A(NM_021140)	KIF5B(NM_004521)	KLLN(NM_001126049)	KMT2D(NM_003482)	KRT5(NM_000424)

L

LARP4(NM_052879)	LBR(NM_002296)	LIG3(NM_013975)	LRIG3(NM_153377)	LRP2(NM_004525)
LATS1(NM_004690)	LGALS8(NM_006499)	LIG4(NM_002312)	LRMDA(NM_032024.3)	LYN(NM_002350)
LATS2(NM_014572)	LGR5(NM_003667)	LMO1(NM_002315)	LRP1B(NM_018557)	LZTR1(NM_006767)

M

MAD1L1(NM_003550)	MAPK3(NM_001040056)	MEN1(NM_000244)	MPL(NM_005373)	MUTYH(NM_001128425)
MALAT1(NR_002819)	MAPKBP1(NM_014994)	MET(NM_000245)	MRE11A(NM_005590)	MYC(NM_002467)
MAP2K1(NM_002755)	MAX(NM_002382)	MGAT4A(NM_012214)	MSH2(NM_000251)	MYCL(NM_001033082)
MAP2K2(NM_030662)	MCL1(NM_021960)	MIR3936HG(NR_110997)	MSH3(NM_002439)	MYCN(NM_005378)
MAP2K4(NM_003010)	MDH2(NM_005918)	MIR4713HG(NR_146310)	MSH6(NM_000179)	MYD88(NM_001172567)
MAP3K1(NM_005921)	MDM2(NM_002392)	MITF(NM_000248)	MST1R(NM_001244937)	MYOD1(NM_002478)
MAP3K6(NM_004672)	MDM4(NM_002393)	MKI67(NM_002417)	MTHFR(NM_005957)	
MAP4K4(NM_004834)	MED12(NM_005120)	MLH1(NM_000249)	MTOR(NM_004958)	
MAPK1(NM_002745)	MEF2B(NM_005919)	MLH3(NM_001040108)	MTUS1(NM_020749)	

N-O

NAB2(NM_005967)	NF2(NM_000268)	NOTCH3(NM_000435)	NRAS(NM_002524)	NTRK3(NM_001007156)
NAT2(NM_000015)	NFE2L2(NM_006164)	NOVA1(NM_006491)	NRG1(NM_001159995)	NUP93(NM_014669)
NBN(NM_002485)	NFKBIA(NM_020529)	NPM1(NM_002520)	NSD1(NM_022455)	NUTM1(NM_175741)
NCOA1(NM_003743)	NKX2-1(NM_003317)	NQO1(NM_000903)	NT5C2(NM_012229)	OPRM1(NM_001008503)
NCOA3(NM_006534)	NOS3(NM_000603)	NQO2(NM_000904)	NTHL1(NM_002528)	OTOS(NM_148961)
NCOA4(NM_001145260)	NOTCH1(NM_017617)	NR1I2(NM_003889)	NTRK1(NM_002529)	
NF1(NM_000267)	NOTCH2(NM_024408)	NR4A3(NM_006981)	NTRK2(NM_006180)	

P

PAK1(NM_002576)	PCLO(NM_014510)	PIK3CG(NM_002649)	POR(NM_000941)	PRSS1(NM_002769)
PALB2(NM_024675)	PDCD1(NM_005018)	PIK3R1(NM_181523)	PPIB(NM_000942)	PSME2(NM_002818)
PALLD(NM_016081)	PDCD1LG2(NM_025239)	PIK3R2(NM_005027)	PPP2R1A(NM_014225)	PTCH1(NM_000264)
PARD3B(NM_057177)	PDGFB(NM_002608)	PIM1(NM_001243186)	PPP2R2A(NM_002717)	PTCH2(NM_003738)
PARK2(NM_004562)	PDGFRA(NM_006206)	PLAUR(NM_002659)	PPP4R4(NM_058237)	PTEN(NM_000314)
PARK7(NM_007262)	PDGFRB(NM_002609)	PLCG2(NM_002661)	PRDM1(NM_001198)	PTGER4(NM_000958)
PARP1(NM_001618)	PDPK1(NM_002613)	PLIN2(NM_001122)	PRDX4(NM_006406)	PTGES(NM_004878)
PAX3(NM_000438)	PGR(NM_000926)	PMS1(NM_000534)	PREX2(NM_025170)	PTGS2(NM_000963)
PAX5(NM_016734)	PIAS4(NM_015897)	PMS2(NM_000535)	PRKACA(NM_002730)	PTN(NM_002825)
PAX7(NM_002584)	PIGB(NM_004855)	POLD1(NM_002691)	PRKACB(NM_182948)	PTPN11(NM_002834)
PAX8(NM_003466)	PIK3CA(NM_006218)	POLE(NM_006231)	PRKAR1A(NM_002734)	PTPRD(NM_001040712)
PBRM1(NM_018165)	PIK3CB(NM_006219)	POLH(NM_006502)	PRKCI(NM_002740)	PTPRT(NM_007050)
PCBP1(NM_006196)	PIK3CD(NM_005026)	POLQ(NM_199420)	PRKDC(NM_006904)	PZP(NM_002864)

R

RAC1(NM_018890)	RAF1(NM_002880)	RET(NM_020630)	RIF1(NM_001177663)	RPS20(NM_001023)
RAD21(NM_006265)	RARA(NM_000964)	REV3L(NM_002912)	RILP(NM_031430)	RPS6KB1(NM_003161)
RAD50(NM_005732)	RB1(NM_000321)	RFC1(NM_002913)	RINT1(NM_021930)	RPTOR(NM_020761)
RAD51(NM_002875)	RBFOX1(NM_145891)	RFC4(NM_002916)	RIT1(NM_006912)	RRAS2(NM_001177314)
RAD51B(NM_002877)	RBM10(NM_005676)	RGS5(NM_003617)	RNASEL(NM_021133)	RRM1(NM_001033)
RAD51C(NM_058216)	RECK(NM_021111)	RHBDF2(NM_024599)	RNF43(NM_017763)	RSF1(NM_016578)

RAD51D(NM_002878)	*RECQL*(NM_002907)	*RHEB*(NM_005614)	*ROBO2*(NM_002942)	*RUNX1*(NM_001754)
RAD52(NM_134424)	*RECQL4*(NM_004260)	*RHOA*(NM_001664)	*ROS1*(NM_002944)	
RAD54L(NM_003579)	*REL*(NM_002908)	*RICTOR*(NM_152756)	*RPA4*(NM_013347)	
		**S**		
SBDS(NM_016038)	*SETD2*(NM_014159)	*SLC22A5*(NM_003060)	*SMARCB1*(NM_003073)	*SRD5A2*(NM_000348)
SCN10A(NM_006514)	*SETD7*(NM_030648)	*SLC28A1*(NM_004213)	*SMO*(NM_005631)	*SRSF2*(NM_003016)
SDHA(NM_004168)	*SF3B1*(NM_012433)	*SLC28A2*(NM_004212)	*SOCS1*(NM_003745)	*SS18*(NM_001007559)
SDHAF2(NM_017841)	*SH2B3*(NM_005475)	*SLC29A1*(NM_004955.2)	*SOCS6*(NM_004232)	*STAG2*(NM_001042749)
SDHB(NM_003000)	*SHMT1*(NM_004169)	*SLC31A1*(NM_001859)	*SOD2*(NM_001024465)	*STAT3*(NM_139276)
SDHC(NM_003001)	*SHOX*(NM_000451)	*SLC34A2*(NM_006424)	*SOX10*(NM_006941)	*STK11*(NM_000455)
SDHD(NM_003002)	*SHPRH*(NM_173082)	*SLCO1B1*(NM_006446)	*SOX2*(NM_003106)	*SUFU*(NM_016169)
SELE(NM_000450)	*SLC15A2*(NM_021082)	*SLCO1B3*(NM_019844)	*SOX4*(NM_003107)	*SULT1A1*(NM_001055)
SELL(NM_000655)	*SLC19A1*(NM_030582)	*SLX4*(NM_032444)	*SOX9*(NM_000346)	*SUZ12*(NM_015355)
SEMA3C(NM_006379)	*SLC22A1*(NM_003057)	*SMAD2*(NM_005901)	*SPEN*(NM_015001)	*SWI5*(NM_001040011)
SERPINB3(NM_006919)	*SLC22A16*(NM_033125)	*SMAD3*(NM_005902)	*SPINK1*(NM_003122)	*SYK*(NM_001135052)
SERPINB4(NM_002974)	*SLC22A2*(NM_003058)	*SMAD4*(NM_005359)	*SPOP*(NM_003563)	*SYNE1*(NM_033071)
SETBP1(NM_015559)	*SLC22A4*(NM_003059)	*SMARCA4*(NM_001128849)	*SRC*(NM_198291)	
		**T**		
TACSTD2(NM_002353)	*TFE3*(NM_006521)	*TNFRSF11B*(NM_002546)	*TP53*(NM_000546)	*TSC2*(NM_000548)
TBX3(NM_005996)	*TGFB1*(NM_000660)	*TNFRSF14*(NM_003820)	*TP53BP1*(NM_001141979)	*TSHR*(NM_000369)
TCEB1(NM_005648)	*TGFBR2*(NM_001024847)	*TNFRSF19*(NM_018647)	*TPMT*(NM_000367)	*TSPAN31*(NM_005981)
TERC(NR_001566)	*TMEM127*(NM_017849)	*TNFSF11*(NM_033012)	*TRAF1*(NM_005658)	*TTK*(NM_001166691)
TERT(NM_198253)	*TMPRSS2*(NM_001135099)	*TNFSF8*(NM_001244)	*TRAM2-AS1*(NR_103446)	*TUBB1*(NM_030773)
TET1(NM_030625)	*TNF*(NM_000594)	*TOP1*(NM_003286)	*TREX2*(NM_080701)	*TYMS*(NM_001071)
TET2(NM_017628)	*TNFAIP3*(NM_006290)	*TOP2A*(NM_001067)	*TSC1*(NM_000368)	*U2AF1*(NM_006758)
		**U-Z**		
UBE2A(NM_003336)	*UBE2I*(NM_003345)	*UBE2V2*(NM_003350)	*UGT1A1*(NM_000463)	*UGT1A4*(NM_007120)
UGT1A6(NM_001072)	*UGT1A9*(NM_021027)	*VEGFA*(NM_001025366)	*VEGFC*(NM_005429)	*VHL*(NM_000551)
WAS(NM_000377)	*WIF1*(NM_007191)	*WNK2*(NM_006648)	*WNT5B*(NM_032642)	*WRN*(NM_000553)
WT1(NM_024426)	*XBP1*(NM_005080)	*XPA*(NM_000380)	*XPC*(NM_004628)	*XPO1*(NM_003400)
XRCC1(NM_006297)	*XRCC2*(NM_005431)	*XRCC4*(NM_022406)	*YAP1*(NM_006106)	*YES1*(NM_005433)
ZBTB16(NM_006006)	*ZNF367*(NM_153695)	*ZNF423*(NM_001271620)	*ZNF750*(NM_024702)	*ZRSR2*(NM_005089)

List of Fusion gene (37)

ALK(NM_004304)	*FGFR1*(NM_023110)	*NAB2*(NM_005967)	*NTRK3*(NM_001007156)	*RARA*(NM_000964)
BRAF(NM_004333)	*FGFR2*(NM_000141)	*NCOA4*(NM_001145260)	*NUTM1*(NM_175741)	*RET*(NM_020630)
CCDC6(NM_005436)	*FGFR3*(NM_000142)	*NPM1*(NM_002520)	*PAX3*(NM_000438)	*ROS1*(NM_002944)
CD74(NM_001025159)	*FUS*(NM_004960)	*NR4A3*(NM_006981)	*PAX7*(NM_002584)	*SLC34A2*(NM_006424)
DDIT3(NM_004083)	*KIF5B*(NM_004521)	*NRG1*(NM_001159995)	*PAX8*(NM_003466)	*SS18*(NM_001007559)
EML4(NM_019063)	*KMT2A*(NM_001197104)	*NTRK1*(NM_002529)	*PDGFB*(NM_002608)	*TFE3*(NM_006521)
EWSR1(NM_005243)	*MYC*(NM_002467)	*NTRK2*(NM_006180)	*PRKACA*(NM_002730)	*TMPRSS2*(NM_001135099)
RELA(NM_021975)	*ERG*(NM_182918)			

List of hereditary tumor-related genes (89)

AIP(NM_003977)	*CDKN2A*(NM_000077)	*HOXB13*(NM_006361)	*POLD1*(NM_002691)	*SDHB*(NM_003000)
ALK(NM_004304)	*CFTR*(NM_000492)	*MAX*(NM_002382)	*POLE*(NM_006231)	*SDHC*(NM_003001)
APC(NM_000038)	*CHEK2*(NM_007194)	*MDH2*(NM_005918)	*PRKAR1A*(NM_002734)	*SDHD*(NM_003002)
ATM(NM_000051)	*CTNNA1*(NM_001903)	*MEN1*(NM_000244)	*PRSS1*(NM_002769)	*SLX4*(NM_032444)
ATR(NM_001184)	*DICER1*(NM_177438)	*MET*(NM_001127500)	*PTCH1*(NM_000264)	*SMAD4*(NM_005359)
AXIN2(NM_004655)	*EPAS1*(NM_001430)	*MLH1*(NM_000249)	*PTEN*(NM_000314)	*SMARCA4*(NM_001128849)
BAP1(NM_004656)	*EPCAM*(NM_002354)	*MLH3*(NM_001040108)	*RAD50*(NM_005732)	*SMARCB1*(NM_003073)
BARD1(NM_000465)	*EXT1*(NM_000127)	*MRE11A*(NM_005590)	*RAD51C*(NM_058216)	*SPINK1*(NM_003122)
BLM(NM_000057)	*EXT2*(NM_000401)	*MSH2*(NM_000251)	*RAD51D*(NM_002878)	*STK11*(NM_000455)
BMPR1A(NM_004329)	*FAM175A*(NM_139076)	*MSH3*(NM_002439)	*RB1*(NM_000321)	*SUFU*(NM_016169)
BRCA1(NM_007294)	*FANCA*(NM_000135)	*MSH6*(NM_000179)	*RECQL4*(NM_004260)	*TERT*(NM_198253)
BRCA2(NM_000059)	*FANCC*(NM_000136)	*MUTYH*(NM_001128425)	*RET*(NM_020630)	*TMEM127*(NM_017849)
BRIP1(NM_032043)	*FANCG*(NM_004629)	*NBN*(NM_002485)	*RHBDF2*(NM_024599)	*TP53*(NM_000546)
CDC73(NM_024529)	*FH*(NM_000143)	*NF1*(NM_000267)	*RINT1*(NM_021930)	*TSC1*(NM_000368)
CDH1(NM_004360)	*FLCN*(NM_144997)	*NF2*(NM_000268)	*RNF43*(NM_017763)	*TSC2*(NM_000548)
CDK12(NM_016507)	*GALNT12*(NM_024642)	*NTHL1*(NM_002528)	*RPS20*(NM_001023)	*VHL*(NM_000551)
CDK4(NM_000075)	*GEN1*(NM_182625)	*PALB2*(NM_024675)	*SDHA*(NM_004168)	*XRCC2*(NM_005431)
CDKN1B(NM_004064)	*GREM1*(NM_013372)	*PMS2*(NM_000535)	*SDHAF2*(NM_017841)	

List of genes associated with DNA damage repair (DDR) (65)

HRR(27):				
ATM(NM_000051)	*CHEK1*(NM_001274)	*FANCI*(NM_001113378)	*PALB2*(NM_024675)	*RAD52*(NM_134424)
ATR(NM_001184)	*CHEK2*(NM_007194)	*FANCL*(NM_018062)	*RAD50*(NM_005732)	*RAD54L*(NM_003579)
BRCA1(NM_007294)	*FANCA*(NM_000135)	*FANCM*(NM_020937)	*RAD51*(NM_002875)	*BARD1*(NM_000465)
BRCA2(NM_000059)	*FANCB*(NM_001018113)	*GEN1*(NM_182625)	*RAD51B*(NM_002877)	
BRIP1(NM_032043)	*FANCC*(NM_000136)	*MRE11A*(NM_005590)	*RAD51C*(NM_058216)	
CDK12(NM_016507)	*FANCG*(NM_004629)	*NBN*(NM_002485)	*RAD51D*(NM_002878)	
MMR(7):				
MLH1(NM_000249)	*MLH3*(NM_001040108)	*MSH2*(NM_000251)	*MSH3*(NM_002439)	*MSH6*(NM_000179)
PMS1(NM_000534)	*PMS2*(NM_000535)			
NER(6):				
ERCC1(NM_001983)	*ERCC2*(NM_000400)	*ERCC3*(NM_000122)	*ERCC4*(NM_005236)	*ERCC5*(NM_000123)
ERCC6(NM_000124)				
DDR(25):				
ATRIP(NM_130384)	*MUTYH*(NM_001128425)	*PRKDC*(NM_006904)	*RPA4*(NM_013347)	*UBE2A*(NM_003336)
BLM(NM_000057)	*PARP1*(NM_001618)	*RECQL*(NM_002907)	*SHPRH*(NM_173082)	*UBE2V2*(NM_003350)
CLK2(NM_003993)	*POLD1*(NM_002691)	*RECQL4*(NM_004260)	*TP53*(NM_000546)	*WRN*(NM_000553)
IDH1(NM_005896)	*POLE*(NM_006231)	*REV3L*(NM_002912)	*TP53BP1*(NM_001141979)	*PTEN*(NM_000314)
LIG4(NM_002312)	*POLQ*(NM_199420)	*RIF1*(NM_001177663)	*TREX2*(NM_080701)	*STAG2*(NM_001042749)

DDR: DNA damage response; **HRR**: Homologous Recombination Repair ;**MMR**: DNA mismatch repair; **NER**: Nucleotide excision repair.

List of virus

EBV-WT	HBV-G	HBV-B	HBV-F	HBV-E
HBV-A	HBV-H	HBV-D	HBV-C	HPV-16
HPV-18	HPV-58	HPV-52	HPV-33	HPV-31
HPV-45	HPV-6b	HPV-11		
